# Aptamers Binding to c-Met Inhibiting Tumor Cell Migration

**DOI:** 10.1371/journal.pone.0142412

**Published:** 2015-12-11

**Authors:** Birgit Piater, Achim Doerner, Ralf Guenther, Harald Kolmar, Bjoern Hock

**Affiliations:** 1 Protein Engineering and Antibody Technologies, Merck KGaA, Darmstadt, Germany; 2 Institute for Organic Chemistry and Biochemistry, Technische Universität Darmstadt, Darmstadt, Germany; Consiglio Nazionale delle Ricerche (CNR), ITALY

## Abstract

The human receptor tyrosine kinase c-Met plays an important role in the control of critical cellular processes. Since c-Met is frequently over expressed or deregulated in human malignancies, blocking its activation is of special interest for therapy. In normal conditions, the c-Met receptor is activated by its bivalent ligand hepatocyte growth factor (HGF). Also bivalent antibodies can activate the receptor by cross linking, limiting therapeutic applications. We report the generation of the RNA aptamer CLN64 containing 2’-fluoro pyrimidine modifications by systematic evolution of ligands by exponential enrichment (SELEX). CLN64 and a previously described single-stranded DNA (ssDNA) aptamer CLN3 exhibited high specificities and affinities to recombinant and cellular expressed c-Met. Both aptamers effectively inhibited HGF-dependent c-Met activation, signaling and cell migration. We showed that these aptamers did not induce c-Met activation, revealing an advantage over bivalent therapeutic molecules. Both aptamers were shown to bind overlapping epitopes but only CLN3 competed with HGF binding to cMet. In addition to their therapeutic and diagnostic potential, CLN3 and CLN64 aptamers exhibit valuable tools to further understand the structural and functional basis for c-Met activation or inhibition by synthetic ligands and their interplay with HGF binding.

## Introduction

The c-Met receptor, also called hepatocyte growth factor receptor (HGFR) is a member of the receptor tyrosine kinase family [1]. It consists of an extracellular ligand-binding domain and an intracellular kinase domain. The receptor is activated by ligand binding followed by dimerization and phosphorylation within the intracellular kinase domains [2]. Structurally, the extracellular domain is composed of a semaphorin (SEMA) domain, a cystein rich hinge known as plexin, semaphorin and integrin (PSI) domain followed by four immunoglobulin-like domains, named after their presence in plexins and transcription factors (IPT) [3,4]. In humans, HGF is the only known activating ligand of c-Met that induces cellular responses such as cell proliferation, cell survival, cell motility and invasion [5–9]. HGF was long known as bivalent factor consisting of α- and β-chain that is activated by proteolytic cleavage [10]. While the binding site of the HGF β-chain was solved by crystal structure [4], the location of the α-chain high affinity binding site still remains a suggestion. Some publications located the binding site of the α-chain in the SEMA domain [11–14], others suggest that its binding site is within the IPT domains [15]. Until now, the exact mechanism of c-Met receptor activation by HGF binding remains unknown.

In healthy tissues, c-Met signaling is implicated in embryonic development [16,17], wound healing and liver regeneration [18,19]. In human malignancies, c-Met can be deregulated by protein overexpression [20,21], gene amplification [22,23], somatic or germline mutations [24,25], or the production of HGF-dependent autocrine loops [26–28]. Several therapeutic concepts aim on inhibiting c-Met signaling in cancer cells such as small molecules, antibodies or decoys of the c-Met extracellular domain (ECD) [29]. Antibodies reducing tumor growths either block HGF binding to c-Met [13,14] or induce shedding followed by down-regulation of the receptor [30,31]. Conventional bivalent antibodies were previously reported to activate the receptor by cross-linking [32]. Attempts in generating monovalent antibody formats succeeded in abolishing the c-Met activating properties of bivalent antibodies [14,30].

Aptamers typically are monovalent binders consisting of a single stranded nucleic acid backbone forming a robust three-dimensional structure. They are usually generated by an *in vitro* selection procedure called SELEX. During the SELEX process, target-binding aptamers are successively enriched by consecutive rounds of selection and amplification [33–35]. Additionally, nucleotide modifications (2’-O-methyl, 2’-fluoro) can be introduced during the amplification of aptamers. Such modifications reduce the susceptibility to nuclease degradation and can improve stability properties [36–39]. Typical dissociation constants (K_D_) of aptamers are in the picomolar to nanomolar range and hence comparable with those of antibodies. The ability of aptamers for receptor-ligand inhibition potentially enables therapeutic applications. Advantages of aptamers over protein-based therapeutics can be cost-effective and uniform synthesis and no to low immunogenicity of the scaffold [40]. The therapeutic aptamer Macugen was approved for age related macular degeneration (AMD) [41]. Further aptamers with therapeutic potential for metabolic, viral, infection, inflammation and cancer diseases have been discovered [40,42,43].

We previously described the development of the c-Met specific ssDNA aptamer CLN3 that could effectively mediate cell lyses in a bi-specific format [44]. While preparing this manuscript, the effect of CLN3 on cell migration was also reported recently [45]. We additionally report the generation of another c-Met specific aptamer CLN64 with rRfY (2’-ribo purine and 2’-fluoro pyrimidine) composition and its influence on HGF-dependent activation of the c-Met pathway.

## Materials and Methods

### Selection of 2’-fluoro pyrimidine (rRfY) RNA aptamers

The DNA library used for selection comprised a 40-nucleotide random region flanked by constant regions that include the priming sites. The initial library was reverse transcribed from the oligonucleotide library GGAGGGAAAAGTTATCAGGC(N)40GATTAGTTTTGGAGTACTCGCTCC using a Thermoscript RT-PCR System Kit (Invitrogen) and amplified by PCR with the forward primer including the T7 promoter site (underlined) GACTGTAATACGACTCACTATAGGAGGGAAAAGTTATCAGGC and the reverse primer GGAGCGAGTACTCCAAAACTAATC. The 2’-fluoro pyrimidine RNA aptamers were then generated by *in vitro* transcription in a 100 μl reaction containing the Y639F mutant of T7 RNA polymerase [39], 2 mM MgCl_2_, 1.5 mM ATP, GTP, 2´-fluoro-CTP (fCTP), 2´-fluoro-UTP (fUTP), 5x pyrophosphatase (Sigma Aldrich). After an over-night incubation at 32°C, aptamers were eluted from a 20% polyacrylamide 6.5 M urea gel.

During selection a 0.5 M KOH treated 0.45 μm nitrocellulose centrex column (Schleicher & Schuell) was used to physically separate target-bound aptamers from unbound aptamers. In total 16 selection rounds were carried out and the amount of target protein was subsequently decreased from 1 μM whereas the amount of aptamer pool exhibited 1.6 μM in the first selection round and 1 μM constantly in subsequent selection rounds. In later rounds up to 1 mg/ml tRNA was added to increase stringency of selection. Aptamers were incubated with the target protein for 1 h at 37°C in 100 μl DPBS (Dulbecco’s PBS, Gibco, Invitrogen) final volume. Each selection round was initiated with a negative selection against a KOH treated nitrocellulose centrex filter and a counter selection against soluble IgG1-Fc (R&D Systems) followed by the positive selection against the soluble target protein c-Met-Fc (R&D Systems). After the positive selection unbound aptamers were removed from the filter by washing twice with DPBS. Bound aptamers were eluted from the filters with preheated 7 M Urea, 100 mM NaOAc, 3 mM EDTA followed by precipitation and amplification as above and previously described [44]. During amplification the number of PCR-cycles needed to amplify a certain amount of DNA were monitored for each SELEX round and used as means to estimate aptamer enrichment during selection. The derived aptamer pool was used in subsequent selection rounds and the procedure was repeated. The enrichment of c-Met specific aptamers within particular aptamer pools was verified by dotblot analyses. Selected aptamer pools were separated by cloning and the sequences were analyzed as previously described [44].

### Aptamer truncation

Truncated DNA aptamers were chemically synthesized (Eurofins MWG Operon). Truncated versions of RNA aptamer CLN64 were produced by in vitro transcription (as above) with the reverse primers GAGTACTCCAAAACTAATCAACAC, CTCCAAAACTAATCAACACAC, AATCAACACACATCCGCTTG, respectively.

### Dotblot analyses for binding affinity and competition analyses

Binding affinities were determined with the nitrocellulose filter method as described previously [44]. Recombinant mouse c-Met-Fc and human EGFR-Fc (both R&D Systems) served as controls. For competitive binding analyses in a dotblot assay, 900 fmol target protein and 100 fmol ^32^P-labeled aptamer were incubated for 30 min in at 37°C. Then 500–16,000 fmol of non-labeled aptamer was added (5–160 fold molar excess). The following aptamers were used as controls; CLN-F (mRfY, 2’-O-methyl purine and 2’-fluoro pyrimidine) GGAGGGAAAAGTTATCAGGCCGCTAGTTACCAGGTGTAGCTGACCAAGCGGATGTGTGTTGATTAGTTTTGGAGTACTCGCTCC, CLN-X (ssDNA) GGAGGGAAAAGTTATCAGGCATGCGCTAGCCGATCGAATCCCGTAGACATTCCGATCGACGATTAGTTTTGGAGTACTCGCTCC and CLN118 (ssDNA) GGAGGGAAAAGTTATCAGGCATCACGTGGTGGGCAAATAACCGGTTGGGGTGGGTCGAGGGATTAGTTTTGGAGTACTCGCTCC.

### Biolayer Interferometry

Biolayer Interferometry (BLI) measurements were carried out on an Octet Red device (fortéBio/Pall). The biosensor tips were rehydrated in 200 μl Dulbecco`s Phosphate Buffered Saline (DPBS) for 10 min. During measurement, shaking conditions at 37°C and a flow rate of 1,000 rpm were applied. A total sample volume of 200 μl was used for the measurements and samples were diluted in DPBS. Protein A coated biosensor tips were used to immobilize c-Met-Fc (R&D Systems). For measuring triplicates, the tips were regenerated with 100 mM citric acid (pH 3). The following instrument settings were used: baseline in DPBS for 60 sec, load in 5 μg/ml c-Met-Fc for 900 sec, baseline in DPBS for 60 sec, association in serial aptamer dilution for 200 sec, dissociation in DPBS for 900 sec, regeneration in 100 mM citric acid (pH 3) for 300 sec. The buffer-subtracted data was analyzed using octet data analysis software (fortéBio/Pall) and applying the global fitting algorithm with unlinked R_Max_ for 1:1 binding stoichiometry. The binding affinity, as expressed by the K_D_, was calculated based on the association (k_on_) and dissociation (k_dis_) rate constants.

### Cell lines

EBC-1 cells (JCRB) were cultured in MEM Eagle medium (Sigma-Aldrich) with 10% FBS (Invitrogen) and 2 mM L-glutamine (Invitrogen). A549 (ATCC), MDA-MB-453 (provided by Danny R. Welch from the Jake Gittlen Cancer Research Institute) and MDA-MB-231 cells (ATCC) were cultured in DMEM (Gibco, Invitrogen) with 10% FBS. NCI-H441 cells (ATCC) were cultured in RPMI 1640 medium (Invitrogen) with 2.5 g/L D(+)-glucose (Sigma-Aldrich), 10 mM HEPES (Gibco, Invitrogen), 2 mM L-glutamine, 1 mM sodium pyruvate (Gibco, Invitrogen) and 10% FBS. Jurkat E6.1 cells were cultured in RPMI 1640 medium with 2 mM glutamine, 1 mM sodium pyruvate and 10% FBS. Cell lines were cultured at 37°C and 5% CO_2_. Only MDA-MB-231 cells were cultured at 10% CO_2_. The MDA-MB-435 cell line was originally reported as breast cancer cell line. Recent studies provided evidence for MDA-MB-435 being a melanoma cell line [46]. In this study, MDA-MB-435 cells were used as negative control for c-Met binding. Data interpretation in this study is therefore not affected by the reported misidentification of this cell line.

### Flow Cytometry

Aptamers were synthesized with 3’-end biotin modification and PAGE-grade purity at IBA GmbH or Biospring GmbH. 100 pmol of biotinylated aptamer was added to 1 × 10^5^ cells in 100 μl PBS / BSA 1% (w/v) and incubated for 30 min at 4°C. The cells were washed with binding buffer and incubated with 100 μl PBS / BSA 1% (w/v) including streptavidin phycoerythrin (Sigma-Aldrich) (1:10) for 30 min at 4°C. The cells were washed twice and resuspended in 200 μl PBS / BSA 1% (w/v) containing propidium iodide (Invitrogen) (1:200) for exclusion of dead cells for analysis. For data acquisition and analysis the Guava easyCyte flow cytometer and the Guava Express Pro software (Millipore) were used.

### Competition ELISA

100 ng HGF (R&D Systems) was immobilized on a Maxisorp microtiter plate (MTP) in 100 μl PBS overnight at 4°C. The remaining binding sites of the MTP surface were blocked with 200 μl PBS / BSA 3% (w/v). c-Met-Fc 250 ng was pre-incubated with various concentrations of aptamer in a final volume of 100 μl PBS / BSA 1% (w/v). The pre-incubated solutions were then added to the pre-treated wells. c-Met-Fc was detected with a horseradish peroxidase (HRP)-conjugated goat anti-human Fc antibody (1:5,000) (Jackson ImmunoResearch). For detection 100 μl 1step Ultra TMB ELISA reagent (Pierce/Thermo Scientific) was added and the chromogenic reaction was stopped with the addition of 100 μl 2 M sulphuric acid. The spectrometric detection at 450 nm was carried out on a Synergy4 reader with the Gen5 software (BioMek/BeckmanCoulter). The values were analyzed by non-linear regression with the log(inhibitor) vs. response—variable slope equation using GraphPad Prism software.

### c-Met phosphorylation assay

0.3 × 10^6^ A549 or EBC-1 cells were seeded in 24-well cell culture plates, grown over night and serum starved for 18 h. The cells were incubated with various amounts of aptamer for 45 min at 37°C. A549 cells were stimulated with 100 ng/ml HGF for 5 min. The medium was removed and the cells were lysed with 100 μl RIPA cell-lyses buffer for 1 h at 4°C. Lysates were spun for 10 min at 13,000 rpm and prepared for SDS-PAGE. Each sample was loaded on two 4–12% BisTris-Gels followed by immunoblotting to PVDF membranes (Invitrogen) and blocking with PBS BSA 5% (w/v). Primary antibodies included rabbit anti-human c-Met (US Biological), rabbit anti-phosphorylated Met (Tyr1234/1235) (US Biological), rabbit anti-Akt, rabbit anti-phosphorylated Akt (Ser473), rabbit anti-p42/44 Erk1/2 mitogen activated protein kinase, rabbit anti-phosphorylated p42/44 Erk1/2 mitogen activated protein kinase (Thr202/Thr204) and rabbit anti-Cofilin (Cell Signaling). Primary antibodies were used in 1:500 dilutions in TBS / BSA 5% (w/v). A HRP-conjugated goat anti-rabbit antibody (Jackson ImmunoResearch) diluted 1:1,000 in TBS / skim milk 5% (w/v) was used as secondary antibody. The immunoblots were developed with the ECL detection method using the Lumi-Light^PLUS^ Western Blotting Substrate (Roche Applied Science) on a VersaDoc instrument with the Quantitiy One software (Bio-Rad).

### Migration assay

0.4 × 10^6^ NCI-H441 cells were seeded in 24-well cell culture plates at 500 μl per well, grown until confluent and serum starved overnight. Cells were treated with defined concentrations of aptamer in 200 μl starvation medium for 1 h. A gap was introduced into the cell monolayer with a P200 pipette tip. Cells were washed with 200 μl pre-warmed DPBS and starvation medium containing aptamers and 100 ng/ml HGF was added to the cells. After 24 h cells were washed with 100 μl ice-cold DPBS and fixed with 100 μl ice-cold methanol for 10 min. Cells were washed with 100 μl de-ionized water (dH_2_0) and stained with 100 μl crystal violet solution for 25 min. Cells were washed three times with 200 μl dH_2_0 and visualized with a Leica DMIL microscope with 5-fold magnification.

### Matrigel invasion assay

Matrigel invasion assays were performed with the transwell FluoroBlok Tumor Invasion System (BD Biosciences) using 24-well inserts. The plate was re-hydrated and DMEM / FBS 0.2% (v/v) containing 100 ng/ml HGF was added to the lower chamber. In the upper chamber, 5×10^4^ MDA-MB-231 human mamma adeno-carcinoma cells in 400 μl DMEM / FBS 0.2% (v/v) were added together with the aptamer. The plates were incubated for 21 h at 37°C and 5% CO_2_. The bottom part of the transwell insert was stained in calcein AM dissolved at 4 μg/ml in Hank’s Balanced Salt Solution (HBSS) for 1 h at 37°C. Stained cells were measured in a Varioscan Flash fluorescence plate reader (Thermo Scientific) at 485/530 nm (excitation/emission) and a reading time of 100 ms. Data was analyzed with a Student’s t-test using GraphPad Prism. Differences at p < 0.05 were considered significant. In addition, invaded Calcein AM stained cells at the bottom of the transwell membrane were visualized by fluorescence microscopy with 488 nm excitation and 10-fold magnification.

## Results

### Aptamer selection and binding affinity

**Fig 1:**
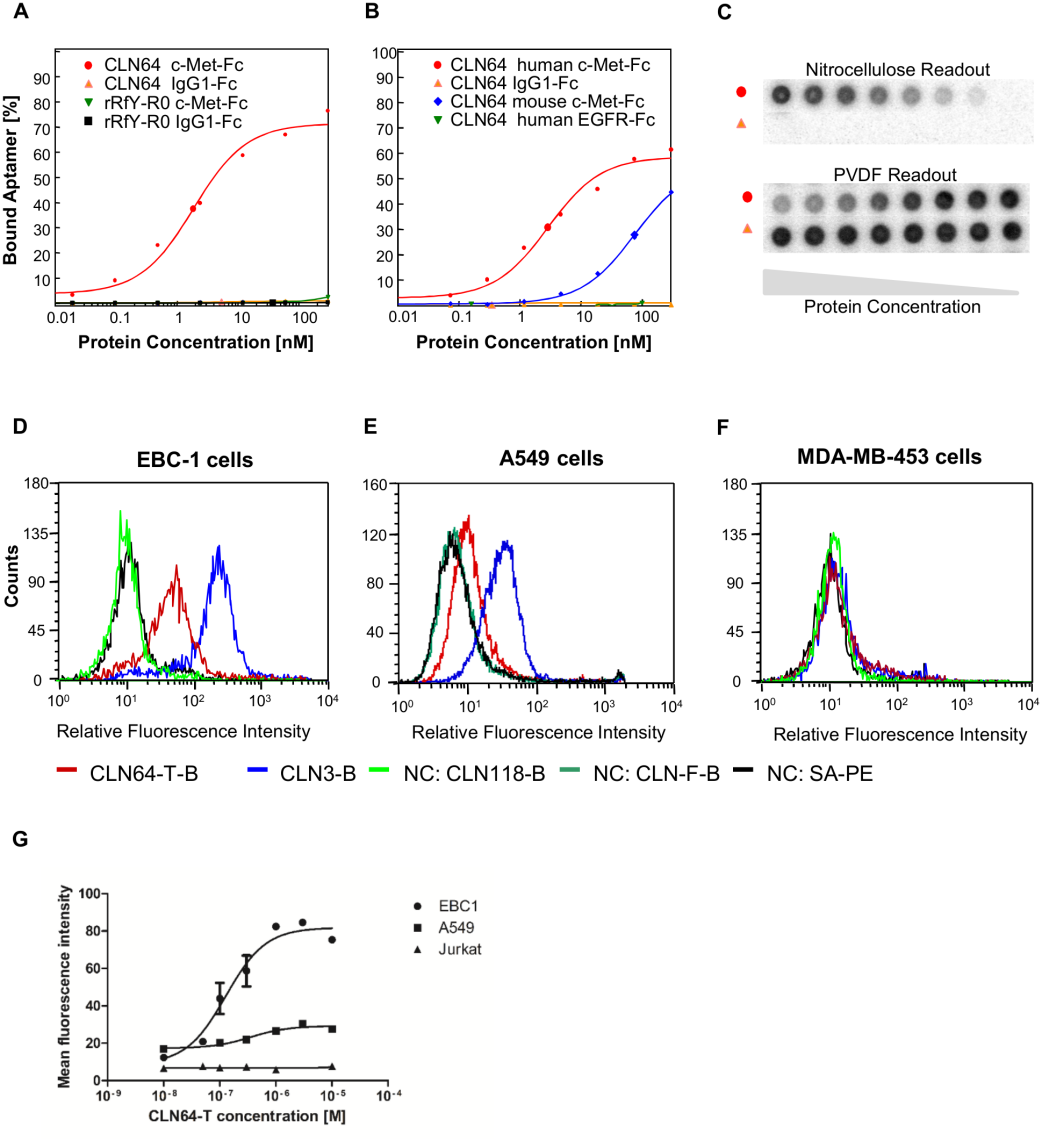
Analyses of the binding properties of CLN64. (A–C) Representative dotblot analyses of the ^32^P-ATP-labeled aptamer CLN64. The unselected rRfY aptamer pool R0, IgG1-Fc and EGFR-Fc served as negative control. Enlarged symbols represent the calculated K_D_ value. (A) Specificity of CLN64 binding to c-Met in comparison to the unselected aptamer pool from 2’-fluoro pyrimidine composition (rRfY) analyzed with dotblot assays. (B) Cross-specificity of CLN64 binding to human c-Met and mouse c-Met by dotblot analyses. (C) Dotblot raw data; nitrocellulose readout for protein-bound CLN64 and PVDF readout for unbound CLN64. (D, E, F) Cellular binding analyses of truncated biotinylated aptamer CLN64 (CLN64-T). Previously described CLN3 was used as reference. CLN118 or CLN-F are unrelated aptamers with unmodified and 2’-fluoro modified compositions and served as negative controls. Biotinylated aptamers were detected with streptavidin phycoerythrin (SA-PE). Binding was tested on (D) EBC-1 cells with high c-Met surface levels, on (E) A549 cells with lower c-Met surface levels and on (F) MDA-MB-453 cells with no detectable c-Met surface levels. MDA-MB-453 cells express the related RON kinase on the cell surface. (G) For determination of the apparent cell surface binding constant, CLN64-T was titrated to EBC-1, A549 and Jurkat E6.1 cells (n = 2).

c-Met specific RNA aptamers with rRfY composition (2’-ribo purine and 2’-fluoro pyrimidine were generated by SELEX with recombinant soluble c-Met-Fc fusion protein as target protein. During the SELEX procedure, selection conditions were constantly increased in stringency by reduction of target protein or addition of competitor tRNA. After 16 selection rounds the affinity of the aptamer pool to c-Met-Fc resulted in a K_D_ value of 7 nM as analyzed by dotblot. Sequence analyses of 96 separated aptamer clones revealed six families with at least 90% sequence identity. These aptamer clones contained only 15% single sequences. One aptamer, CLN64, was highly abundant and accounted for 44% of all analyzed clones (S1 Fig). Initial dotblot binding analyses showed that aptamer CLN64 bound to c-Met-Fc with high affinity (K_D_ = 1 nM). The specificity of this aptamer was analyzed by binding assays with related proteins. CLN64 did not bind to IgG1-Fc and is therefore specific for the c-Met moiety of the fusion protein. Aptamer CLN64 also reacted with lower affinity with murine c-Met. No binding was observed with the receptor tyrosine kinase EGFR (Fig 1A–1C). In conclusion, CLN64 bound to c-Met with high affinity and specificity to the ectodomain. For optimization purposes, the aptamer was truncated for subsequent assays. Truncation by 20 nucleotides at the 3’-end almost completely retained the affinity to c-Met-Fc (S2 Fig). The resulting molecule was designated CLN64-T.

### Cellular binding and aptamer truncation

Highly specific aptamers can fail in binding to the cellular target protein if the selection was carried out with a recombinant target protein which structurally differs from the cell surface protein. For analyzing the binding characteristics to the native cellular receptor, flow cytometry was carried out with human lung cancer cell lines EBC-1, A549 and the human breast cancer cell line MDA-MB-453. Both EBC-1 and A549 cells are positive for c-Met expression, whereas EBC-1 cells express higher surface levels of c-Met than A549 cells. In contrast MDA-MB-453 cells do not express c-Met but RON kinase [47]. The T-cell leukemia cell line Jurkat E6.1 was additionally used as negative control [44]. RON is the second member of the human HGF receptor family and the closest related receptor to c-Met [48,49]. Biotinylated CLN64-T bound to both EBC-1 and A549 cells and not to MDA-MB-453 cells. The measured fluorescence intensities correlated with the different c-Met surface expression levels of these cell lines. To exclude unspecific binding of the nucleic acid backbone to cell surfaces, unrelated aptamers CLN118-B and CLN-F-B with different modifications were used as additional negative controls. None of these control aptamers bound to the cellular surfaces (Fig 1D and 1F). Specificity of CLN64-T was also underlined by achieving binding saturation on EBC-1 and A549 cells whereas and no binding to Jurkat E6.1 cells was observed (Fig 1G). The apparent cell surface binding constants ranged from 100–400 nM. This confirmed high specificity to the cellular c-Met receptor and excluded unspecific cell surface binding. A previously described biotinylated ssDNA aptamer CLN3 with picomolar K_D_ served as reference [44]. In terms of optimization, CLN3 was also truncated at both ends. Truncation of the 84-mer at the 5’-end by five nucleotides and at the 3’-end by 20 nucleotides almost completely retained the binding affinity to c-Met-Fc (K_D_ = 100 pM). The resulting molecule was designated CLN3-T (S2 Fig). Further truncations at the 5’-end were previously shown to shift the affinity towards the nanomolar range [44,45,50]. Biolayer Interferometry (BLI) additionally confirmed the affinities of CLN64-T and CLN3-T to be in the sub-nanomolar to single-digit nanomolar range (S3 Fig).

### Epitope binding and competition with HGF

**Fig 2:**
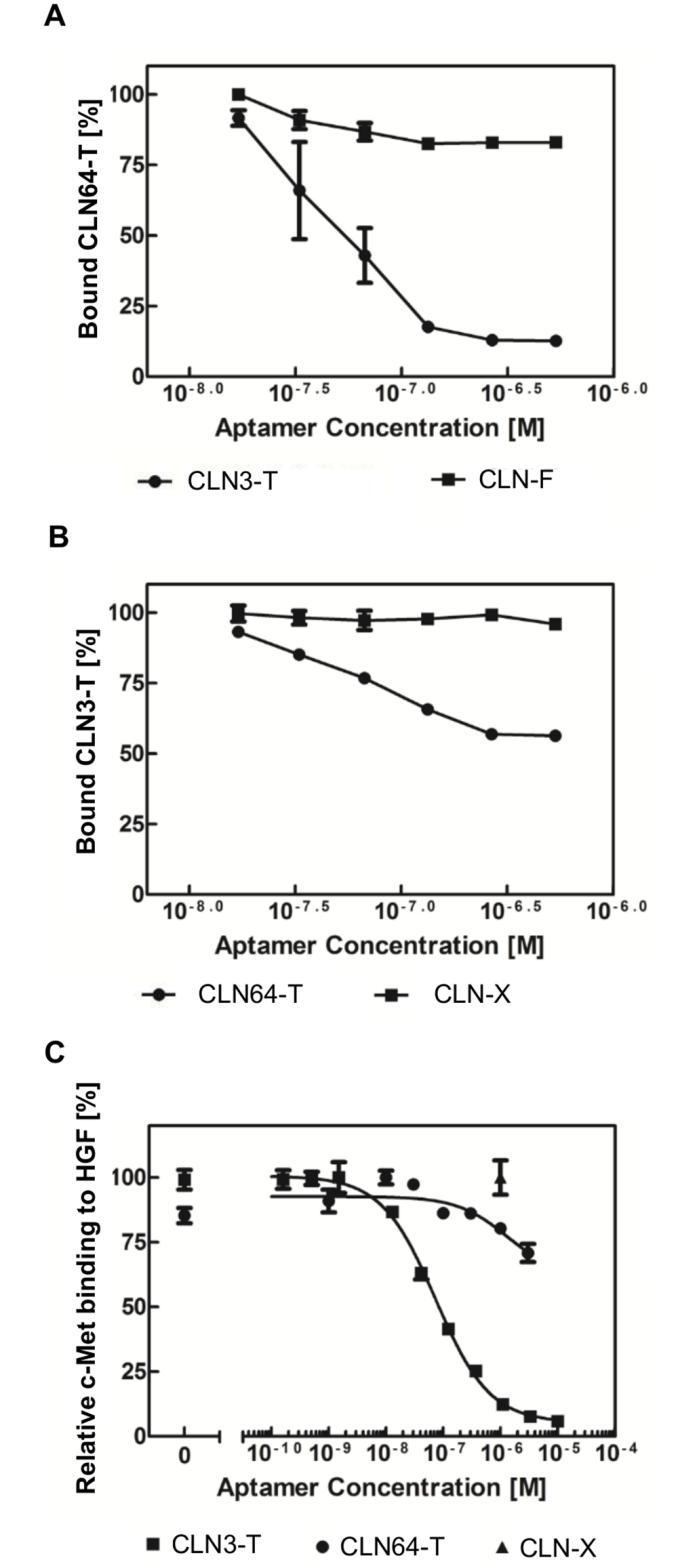
Epitope characterization of CLN64-T and CLN3-T on the c-Met ectodomain. (A, B) Competitive dotblot assays. (A) ^32^P-ATP-labeled CLN64-T was almost completely displaced by 0.6–17.7 fold molar excess of non-labeled CLN3-T and not by the negative control aptamer with 2’-fluoro pyrimidine modifications CLN-F (n = 2). (B) ^32^P-ATP-labeled CLN3-T was partially displaced by non-labeled CLN64-T and not by the negative control ssDNA aptamer CLN-X (n = 2). (C) In a competitive ELISA the microtiterplate-surface was coated with HGF and to this, c-Met-Fc pre-incubated with varying amounts of aptamer, was added. C-Met binding was subsequently analyzed. In a concentration range of 1.6×10–10 and 1×10-5 M, CLN3-T totally inhibited c-Met interaction with HGF at an IC_50_ value of 72 nM. CLN64-T did not totally inhibit HGF binding to c-Met in the given concentration range. A non-cMet binding control ssDNA aptamer CLN-X achieved similar results (n = 2).

CLN64 and CLN3 were derived from different selections and pool compositions (ssDNA and rRfY) and were shown to bind to the c-Met extracellular domain. The epitopes of both aptamers on the c-Met extracellular domain were narrowed down by competition analysis and unrelated aptamers from related compositions served as controls. A competitive nitrocellulose filter assay showed that binding of radio-labeled CLN64-T to c-Met-Fc was almost completely displaced by non-labeled CLN3-T (Fig 2A). Non-labeled CLN64-T also displaced 50% of radio-labeled CLN3-T for c-Met-Fc binding. In both cases a non-binding aptamer did not compete with CLN64-T or CLN3-T for binding to c-Met-Fc. This confirms the specificity of the aptamer displacement (Fig 2A and 2B). Taken together, these analyses indicated that both c-Met-specific aptamers bound overlapping epitopes on the c-Met ectodomain. Functionally important epitopes on the c-Met ectodomain are the ligand binding sites. Binding of HGF to the high and low affinity binding site on the c-Met ectodomain is a prerequisite for receptor activation and signaling. A competitive ELISA showed that CLN3-T blocked HGF binding to c-Met at an IC_50_ of 74 nM. In contrast, CLN64-T did not inhibit HGF binding to c-Met within the tested concentration range. The same result was observed for a non c-Met binding aptamer CLN-X (Fig 2C). Taken together, these results indicate that at least CLN3-T binds in one of the two HGF binding sites within the c-Met ectodomain. The epitope of CLN3-T is at least partially overlapping with CLN64-T.

### Influence on c-Met phosphorylation and signaling

**Fig 3:**
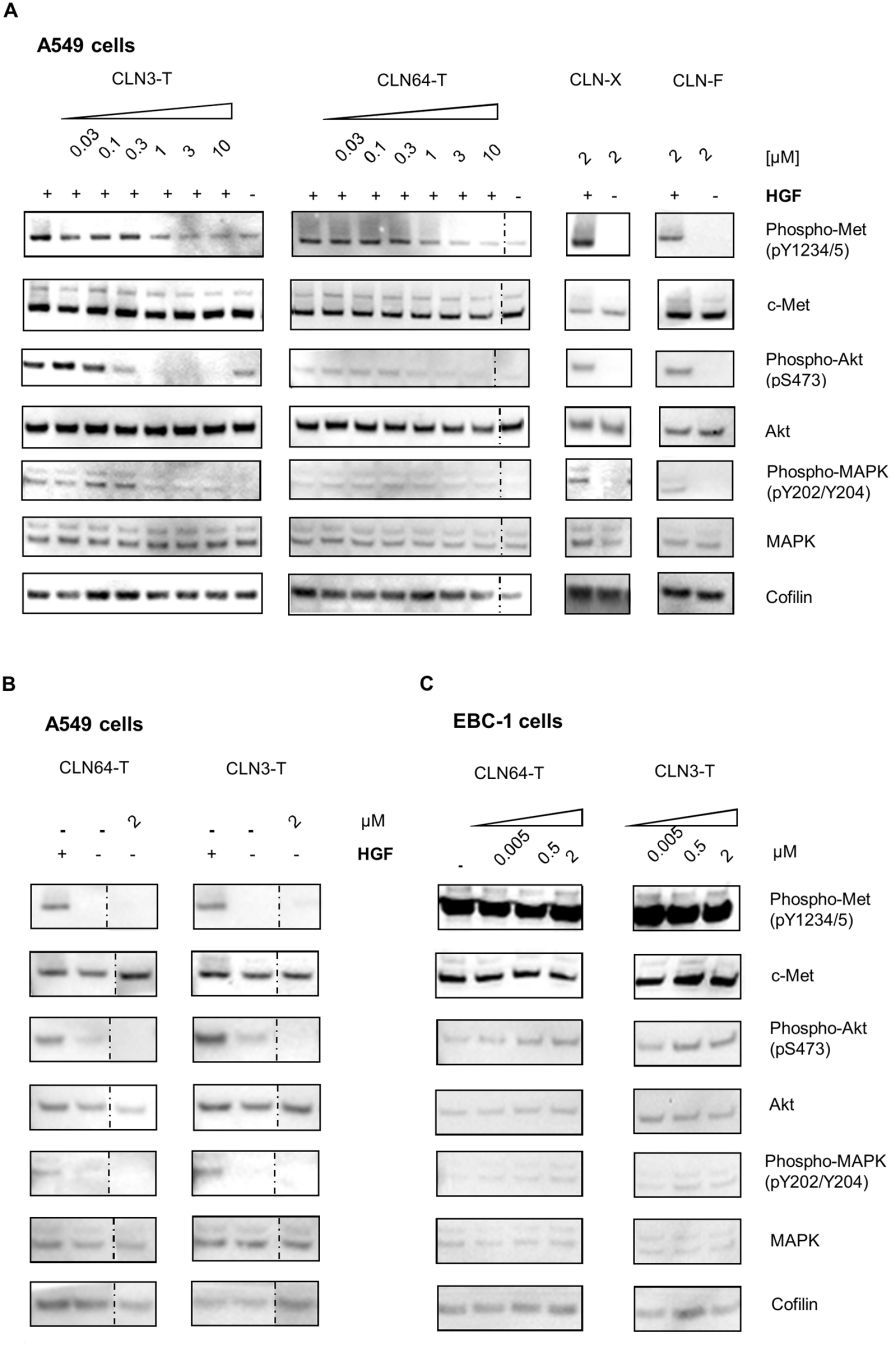
CLN3-T and CLN64-T influence on c-Met phosphorylation and signal transduction in A549 and EBC-1 cells. (A, B) A549 cells or (C) EBC-1 cells were serum-starved and treated with indicated amounts of c-Met-specific CLN3-T, CLN64-T; non-c-Met binding aptamers CLN-X and CLNF. (A, B) A549 cells were additionally stimulated with HGF for 5 min. (A–C) Cell lysates were analyzed by immunoblotting for the availability of phosphorylated c-Met (Phospho-Met), c-Met, phosphorylated Akt (Phospho-Akt), Akt, phosphorylated MAPK (Phospho-MAPK), MAPK and the actin-binding protein Cofilin as loading control. (A) Both aptamers CLN3-T, CLN64-T inhibited HGF-mediated phosphorylation of c-Met and downstream transducers at 3 μM concentrations. No effect was observed for CLN-X and CLN-F. (B) CLN3-T, CLN64-T did not activate c-Met without HGF stimulation. (C) No inhibition of receptor phosphorylation was observed on EBC-1 cells expressing activated c-Met. Dotted lines indicate unconnected spots on the same membrane. The respective anti c-Met and anti Phospho-Met images were derived from two different gels.

Both HGF binding and receptor overexpression induce dimerization, phosphorylation and activation of c-Met. Inhibitory effects on c-Met phosphorylation by admistration of ssDNA and RNA aptamers were thus evaluated in HGF-dependent and HGF-independent cell lines. HGF-dependent c-Met activation was analyzed in A549 cells. In EBC-1 cells c-Met is overexpressed by gene amplification and constitutively phosphorylated [51]. These cells were used to analyze effects on HGF-independent phosphorylation. Downstream signaling pathways activated by c-Met include RAS-RAF-MAPK and PI3K-Akt. Among the signal transducers, MAPK and Akt are activated by phosphorylation. Immunoblotting of stimulated A549 cells showed that CLN3-T and CLN64-T dose dependently inhibited HGF-mediated phosphorylation of c-Met. Complete inhibition was observed at treatment with 1–3 μM of each aptamer. In both cases inhibition of c-Met phosphorylation synergized with the phosphorylation of downstream transducers AKT and MAPK (Fig 3A). Importantly neither CLN3-T nor CLN64-T induced c-Met phosphorylation when administered without HGF-stimulation (Fig 3B). Treatment with non-binding aptamers CLN-X (DNA) and CLN-F (mRfY) neither activated the receptor nor inhibited HGF-mediated receptor activation (Fig 3A and 3B). Furthermore, CLN64-T and CLN3-T did not affect receptor phosphorylation in EBC-1 cells (Fig 3C). In summary, both c-Met-specific aptamers were able to inhibit HGF-induced receptor phosphorylation and subsequent signaling in A549 cells.

### c-Met-dependent cell migration

**Fig 4:**
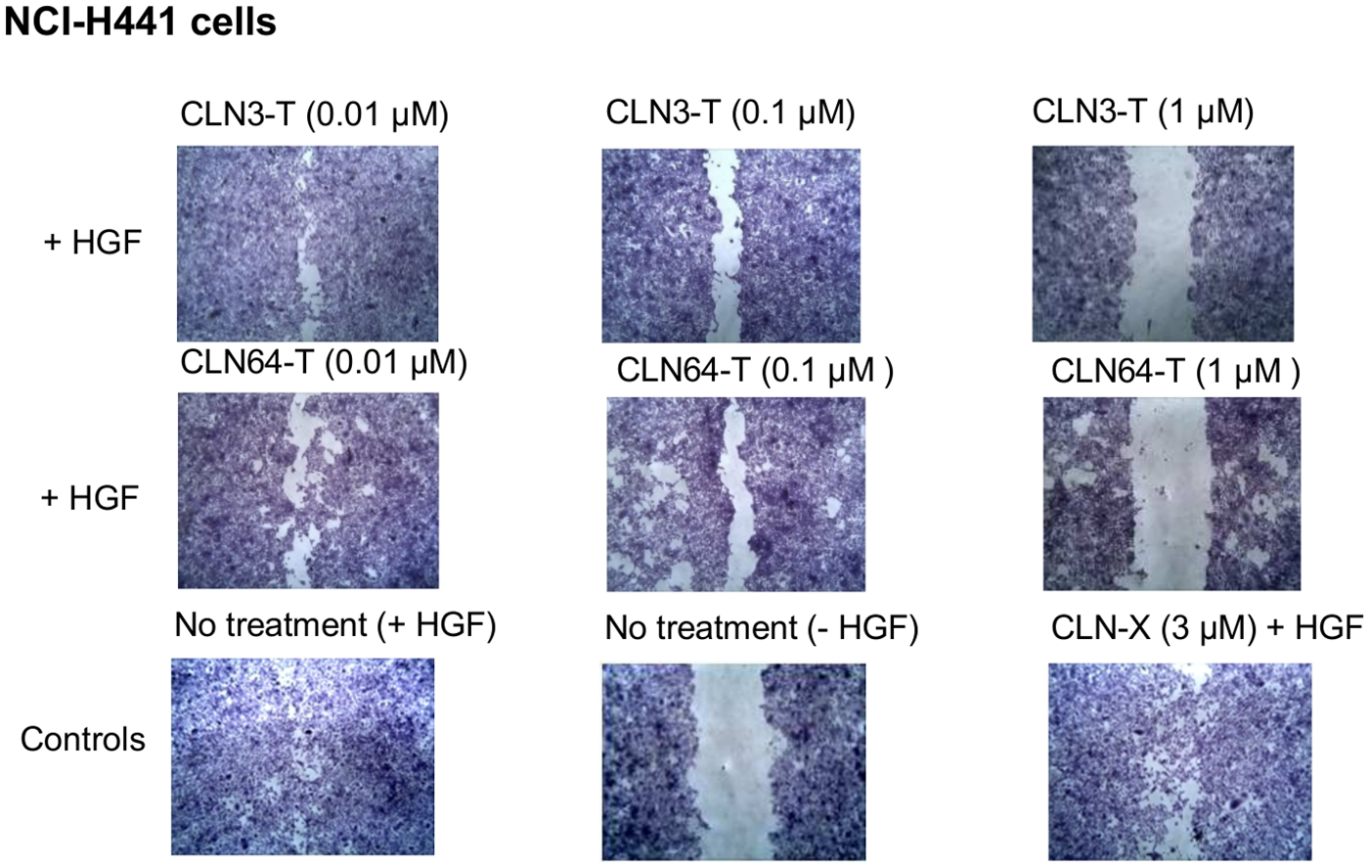
Effects of CLN3-T and CLN64-T on HGF-induced migration on NCI-H441 cells and matrigel invasion of MDA-MB-231 cells. NCI-H441 were grown until confluent and treated with CLN3-T, CLN64-T and CLN-X (negative control) at the indicated concentrations. A gap was introduced and cell migration was stimulated with 100 ng/ml HGF at maintained aptamer treatment. 1 μM of CLN3-T, CLN64-T inhibited HGF-mediated migration of NCI-H441 cells. Treatment of unrelated aptamer CLN-X did not show any effect on cell migration. Representative pictures of two independent experiments are shown.

Activation of c-Met leads to cellular effects such as proliferation, cell survival, motility and invasion [52]. Inhibition of c-Met activation usually correlates with downstream effects. The phenotypic effect of both aptamers, CLN3-T and CLN64-T were evaluated in a migration assay with NCI-H441 human lung papillary adenocarcinoma cells. This cell line exhibits constitutive phosphorylation of c-Met whereas HGF additionally induces c-Src-dependent FAK phosphorylation and cell motility [53]. Both aptamers inhibited HGF-mediated migration of NCI-H441 cells in a dose dependent manner. The non c-Met-binding aptamer CLN-X did not influence migration of NCI-H441 cells (Fig 4). These results are in accordance with the observed HGF-dependent inhibition of receptor phosphorylation.

## Discussion

The c-Met receptor regulates critical pathways involved in cancer development and progression. Therefore, blocking c-Met signaling gained interest for therapeutic approaches. Several biomolecules have been developed that compete with HGF for receptor binding and are active in HGF-responsive cell lines and xenograft models [14,54–56]. So far, only a few molecules are able to inhibit c-Met signaling in HGF-independent models. Among them is the DN30 Fab molecule that acts through receptor shedding [31] and small molecules inhibiting the kinase domain [57]. The main drawback of the kinase inhibitors is reduced target specificity [58].

In this study we describe the selection of an aptamer with affinity to c-Met named CLN64-T. This aptamer was isolated by SELEX from an rRfY modified RNA aptamer pool and therefore can be expected to be resistant against nuclease cleavage. Biochemical characterizations revealed high specificity of CLN64 to recombinant human c-Met ECD and cross-reactivity to the murine counterpart. This indicates that CLN64 binds a structurally conserved region across the human and murine species. Furthermore CLN64-T did not bind to the structurally related RON receptor as shown by FACS analysis. The ECDs of c-Met and RON are very specific for binding their respective ligands HGF and MSP [49,59], concluding that their epitopes are not structurally conserved [60]. Taken together these findings confirm high specificity and selectivity of CLN64-T for the c-Met ECD. This is in accordance with previous findings that aptamers with ability to discriminate between different members of a protein family can be isolated [61–63].

From a biological point of view, c-Met phosphorylation activates complex signaling pathways including Ras-Raf-MAPK and PI3K-Akt. These pathways provoke cellular responses such as cell proliferation, cell survival, cell motility and invasion. The related phenotypes are also involved in tumor initiation and cancer progression [64]. The influence on c-Met receptor activation was analyzed for the truncated version of CLN64 and the previously described aptamer CLN3 [44,45,50]. Both aptamers blocked c-Met receptor activation, downstream signaling and cell migration. Previously, aptamers blocking c-Met activation by binding to the ligand HGF have also been described [65]. The theoretical monovalencies of both aptamers CLN3-T and CLN64-T are comparable with one-armed c-Met binding antibody formats [14]. It is well known that bivalent antibodies activate c-Met [32]. This is why monovalent anti-c-Met antibodies were in clinical trials. It will be interesting to investigate, whether and to what extent bivalent aptamers act as receptor agonists. CLN3-T and CLN64-T did not activate c-Met signaling. This underlines that monovalency is a critical feature for c-Met targeting therapies in cancer. Both aptamers CLN3-T and CLN64-T inhibited HGF-mediated phosphorylation of c-Met in A549 cells. We also showed that inhibition of c-Met signaling by CLN3-T and CLN64-T synergized with the inhibition of migration of NCI-H441 cells. In conclusion, both aptamers blocked activity in HGF-dependent cell lines and not in the HGF-independent cell line EBC-1. This result is consistent with the finding that CLN3 competes with HGF for c-Met binding [45]. Both aptamers bind to the recombinant protein with affinities in the low nanomolar range. Interestingly, the apparent cell surface binding constant of CLN64-T was 100–400 nM. Furthermore an apparent cell surface binding constant of 123 nM was reported for CLN3-T (alias SL1) [45]. It seems that the aptamers bind to the cell surface c-Met with lower affinity than to the purified protein. But the apparent cellular binding constants correlate well with the aptamer amounts needed for functional inhibition (1–3 μM). Interestingly, both aptamers share at least in part the same receptor binding site, whereas only CLN3-T but not CLN64-T is able to inhibit binding of HGF to c-Met. The finding that both aptamers effectively block intracellular signaling indicates that this is mainly due to inhibition of receptor activation. In case of CLN64-T, HGF seems to bind additionally to the receptor but does not trigger receptor activation. This is in accordance with the finding that HGF binding alone, which presumably occurs in the high affinity binding site of c-Met, is necessary for receptor activation but also requires binding to the low affinity binding site in the SEMA domain [15]. Also the HGF precursor pro-HGF binds to c-Met without activating the receptor [10]. There is a lack in the general knowledge of the exact HGF high affinity binding site and the mode of binding for receptor activation. Thus, both aptamers may become valuable tools for the determination of important epitopes for therapeutic approaches targeting c-Met with ramifications for diagnostic and therapeutic applications.

## Supporting Information

S1 Fig SELEX conditions and enrichment for specific aptamers during c-Met-Fc SELEX with the rRfY aptamer pool. (A) Overview of selection conditions for each selection round. In general, 2–3 washing steps were carried out during filter selection and 0–1 mg/ml tRNA were used as nonspecific competitor. (B) Affinities of aptamer pools from rounds R0, R12 and R16 to c-Met-Fc were analyzed with dotblot assays. Magnified symbols indicate calculated K_D_ values. (C) The percentage of sequences within a family of at least 90% sequence identity (percentage of respective single sequences in brackets) in R16 is indicated. One aptamer of each sequence family is shown. The aptamers were grouped by identified motif blocks (black frames). Affinities to the target and counter target protein are also shown in K_D_ values analyzed by dotblot assays.
(TIF)

S2 Fig Truncated variants of CLN64 and CLN3 with overview of sequences and binding data. Binding of CLN64 (A) and CLN3 (B) truncated variants to c-Met-Fc were analyzed using a dotblot assay. The number of truncated nucleotides was indicated by Δx at the respective 5’ or 3’-end. Enlarged symbols represent calculated K_D_ values at equilibrium. The respective tables summarize K_D_ values with standard deviations for n repetitions of the particular experiment. The K_D_ value of CLN3 was determined in [44] and is marked with asterisk. Sequences of truncated CLN64 (A) and CLN3 (B) variants are shown. The randomized aptamer region is marked by a black frame. In this study CLN64-3’Δ20 was referred to as CLN64-T and 5'Δ5-CLN3-3'Δ20 was referred to as CLN3-T.
(TIF)

S3 Fig Interaction analyses of CLN64-T and CLN3-T with c-Met-Fc using Biolayer Interferometry. Each aptamer was measured three times in a four or three-membered three-fold dilution series. The graphs show one representative measure thereof. The used aptamer concentrations are listed next to the particular graphs. The noisy lines represent the measured data and smooth lines represent the binding curves from global full fitting analyses. Kinetic parameters (K_D_, k_on_, k_diss_) derived from three measures are summarized in the tables under the respective graphs.
(TIF)

S4 Fig Full range western blot images for band cut outs presented in the manuscript. For each analysis the membrane for the detection of the phosphorylated protein (upper image) and the total protein (lower image) is shown. A549 cells treated with various concentrations of CLN64-T and CLN3-T and the control aptamer CLN-X.
(TIF)

S5 Fig Full range western blot images for band cut outs presented in the manuscript. For each analysis the membrane for the detection of the phosphorylated protein (upper image) and the total protein (lower image) is shown. A549 cells treated with three concentrations of CLN64-T and CLN3-T and the control aptamers CLN-X and CLN-F.
(TIF)

S6 Fig Full range western blot images for band cut outs presented in the manuscript. For each analysis the membrane for the detection of the phosphorylated protein (upper image) and the total protein (lower image) is shown. EBC-1 cells treated with three concentrations of CLN64-T and CLN3-T and the control aptamer CLN-X.
(TIF)

S7 Fig Flow cytometry analyses comparing different c-Met expression levels of used cell lines. EBC-1, A549, NCI-H441, MDA-MB-453, Jurkat E6.1 and MDA-MB-231 cells were analyzed with anti-c-Met antibody at saturating concentrations.
(TIF)

S8 Fig Flow cytometry for analyzing RON expression levels on MDA-MB-453 and EBC-1 cell lines. (TIF)

S9 Fig Flow cytometry analyses of CLN3-T binding to NCI-H441 cells used for the migration assay. In comparison to the manuscript, c-Met specific aptamer CLN3-T and control aptamer CLN-X were directly labeled with ALEXA488. When comparing the signal on EBC-1 cells, the signal amplification with directly labeled ALEXA488 is lower than for detection with biotinylated aptamers and streptavidin phycoerythrin. Nevertheless CLN3-T shows a low signal for binding to NCI-H441 cells, which can likely be amplified by the use of biotinylated aptamers.
(TIF)

S10 Fig Matrigel cell invasion assay with CLN3-T. Matrigel cell invasion was determined by fluorescence measurements 485/530 nm (excitation/emission) (n = 3) at various concentrations of CLN3-T treatment. ** p < 0.05 versus the control group treated with HGF only. The invaded cells were also visualized by fluorescence microscopy (excitation: 488 nm) with a 10-fold objective.
(TIF)
