# Supplementary figures and images for: Aptamers Binding to c-Met Inhibiting Tumor Cell Migration
Source: PLoS One. 2015 Dec 11;10(12):e0142412. doi: 10.1371/journal.pone.0142412 (PMC4676636; doi:10.1371/journal.pone.0142412)

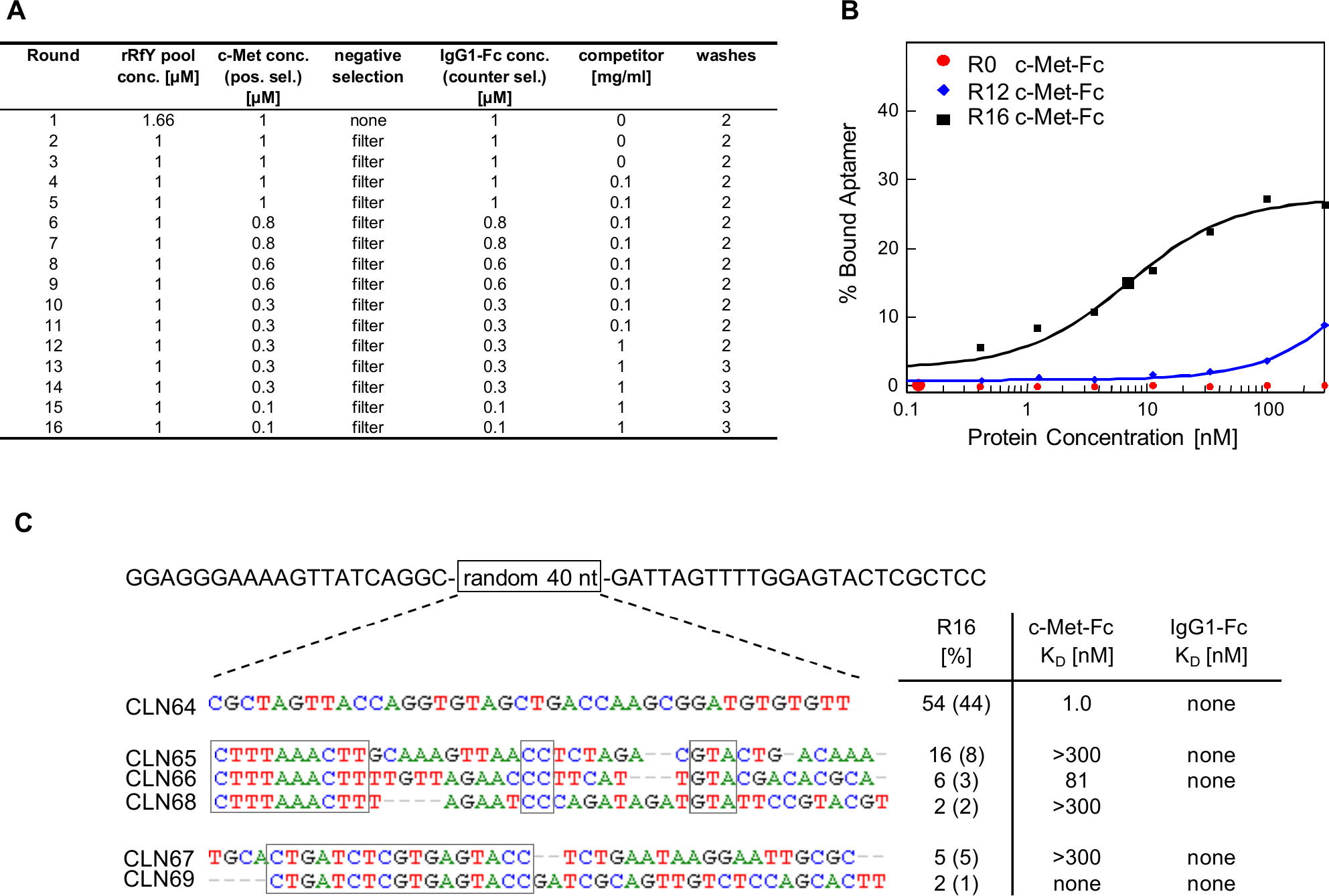

Supplement: S1 Fig — (A) Overview of selection conditions for each selection round. In general, 2–3 washing steps were carried out during filter selection and 0–1 mg/ml tRNA were used as nonspecific competitor. (B) Affinities of aptamer pools from rounds R0, R12 and R16 to c-Met-Fc were analyzed with dotblot assays. Magnified symbols indicate calculated KD values. (C) The percentage of sequences within a family of at least 90% sequence identity (percentage of respective single sequences in brackets) in R16 is indicated. One aptamer of each sequence family is shown. The aptamers were grouped by identified motif blocks (black frames). Affinities to the target and counter target protein are also shown in KD values analyzed by dotblot assays. (TIF) [file pone.0142412.s001.tif]

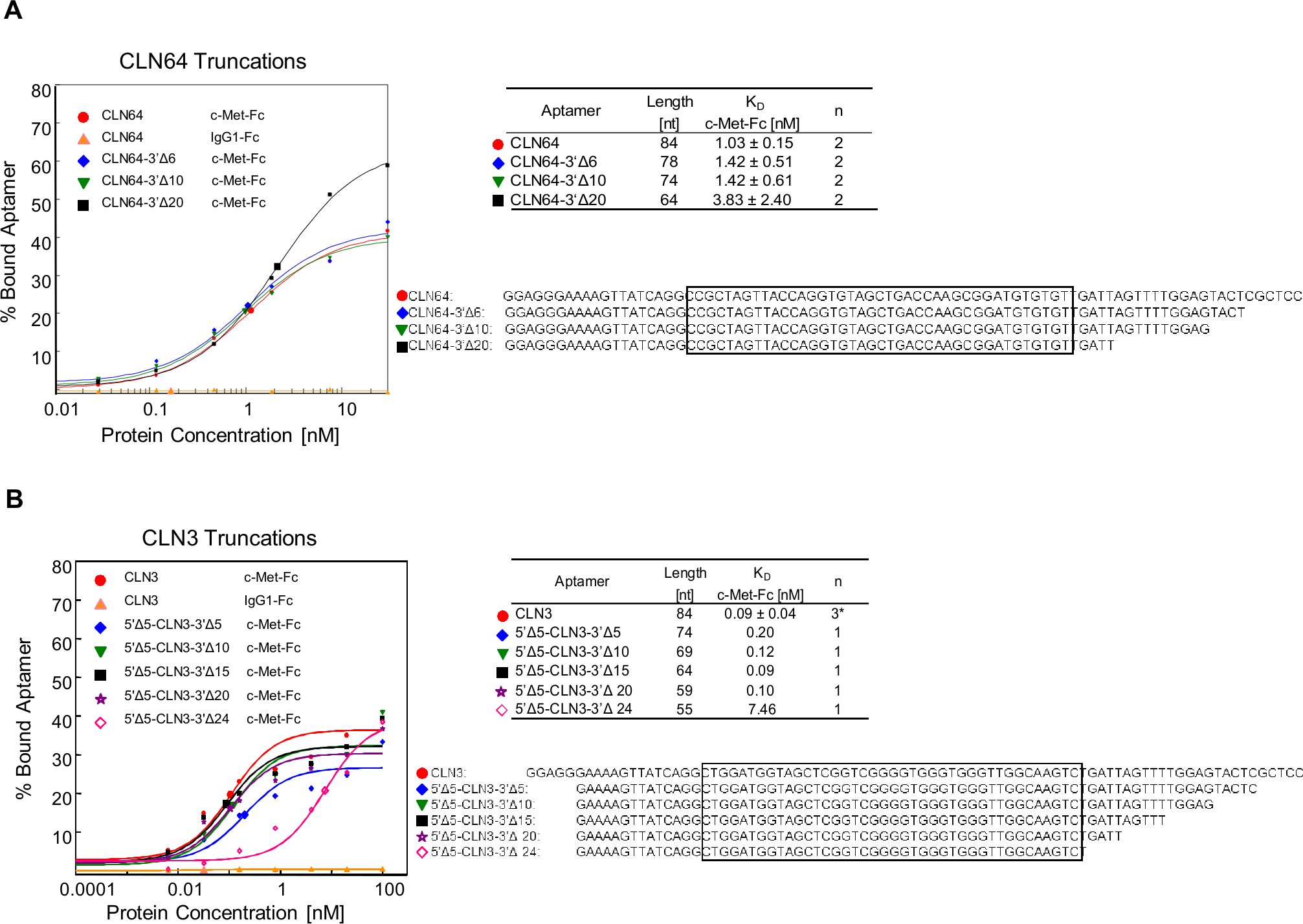

Supplement: S2 Fig — Binding of CLN64 (A) and CLN3 (B) truncated variants to c-Met-Fc were analyzed using a dotblot assay. The number of truncated nucleotides was indicated by Δx at the respective 5’ or 3’-end. Enlarged symbols represent calculated KD values at equilibrium. The respective tables summarize KD values with standard deviations for n repetitions of the particular experiment. The KD value of CLN3 was determined in [44] and is marked with asterisk. Sequences of truncated CLN64 (A) and CLN3 (B) variants are shown. The randomized aptamer region is marked by a black frame. In this study CLN64-3’Δ20 was referred to as CLN64-T and 5'Δ5-CLN3-3'Δ20 was referred to as CLN3-T. (TIF) [file pone.0142412.s002.tif]

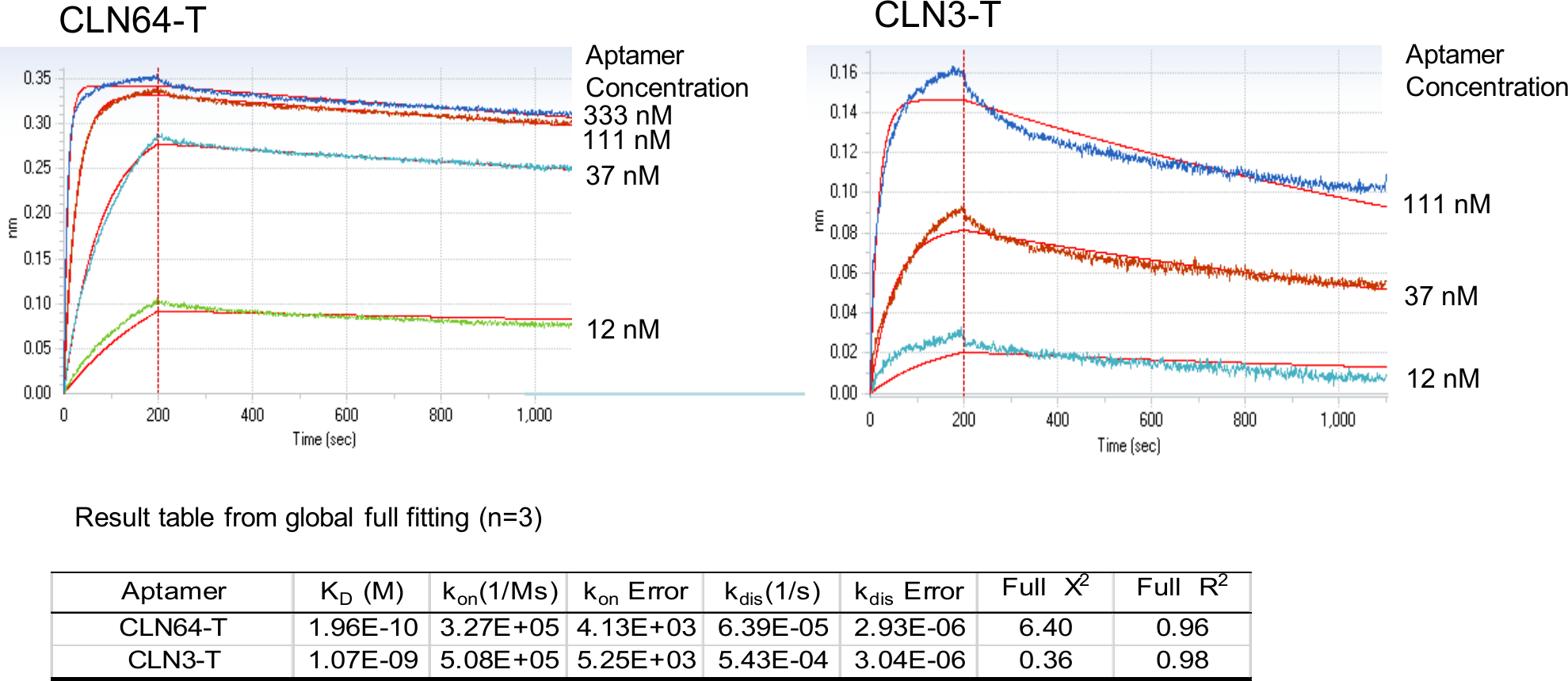

Supplement: S3 Fig — Each aptamer was measured three times in a four or three-membered three-fold dilution series. The graphs show one representative measure thereof. The used aptamer concentrations are listed next to the particular graphs. The noisy lines represent the measured data and smooth lines represent the binding curves from global full fitting analyses. Kinetic parameters (KD, kon, kdiss) derived from three measures are summarized in the tables under the respective graphs. (TIF) [file pone.0142412.s003.tif]

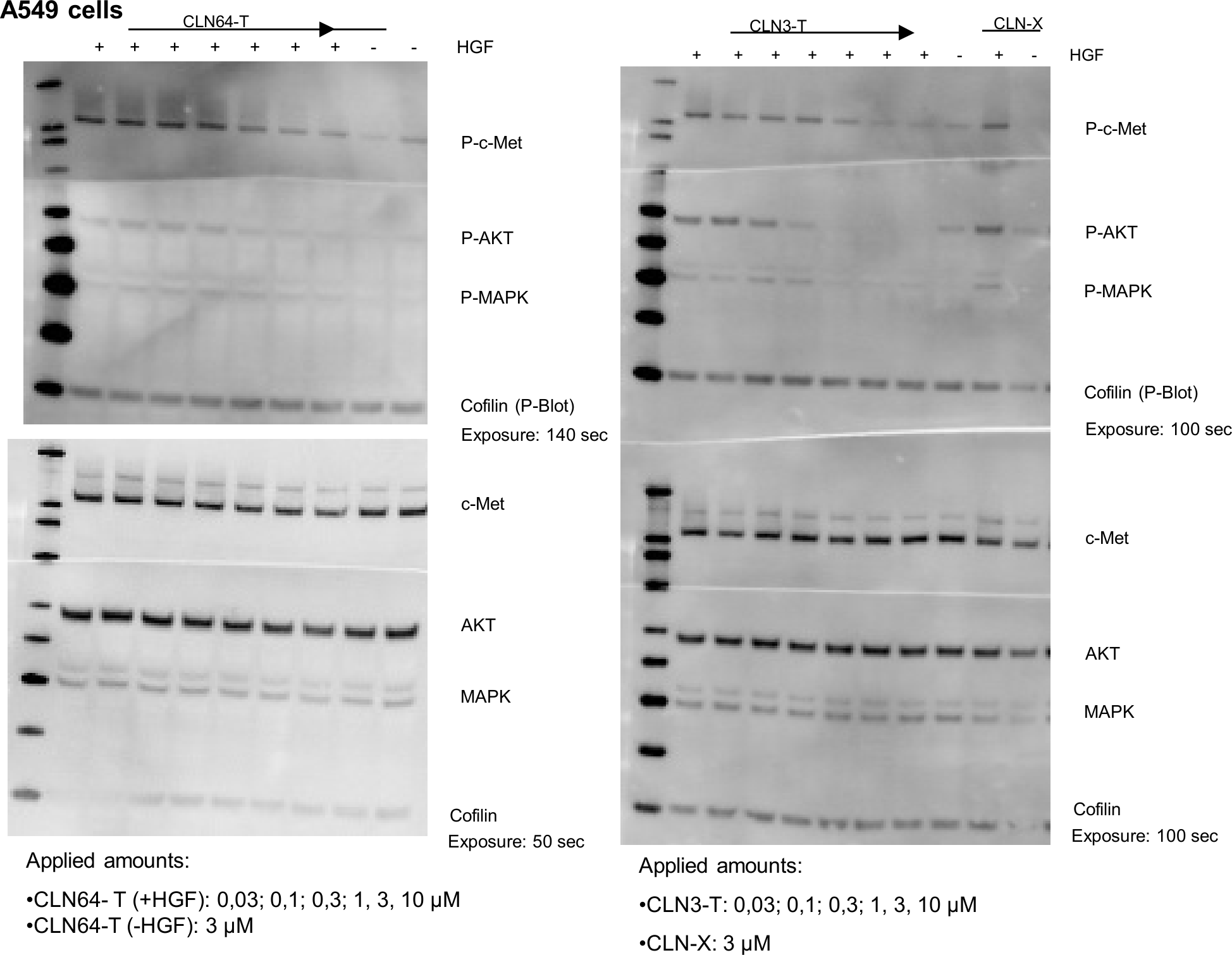

Supplement: S4 Fig — For each analysis the membrane for the detection of the phosphorylated protein (upper image) and the total protein (lower image) is shown. A549 cells treated with various concentrations of CLN64-T and CLN3-T and the control aptamer CLN-X. (TIF) [file pone.0142412.s004.tif]

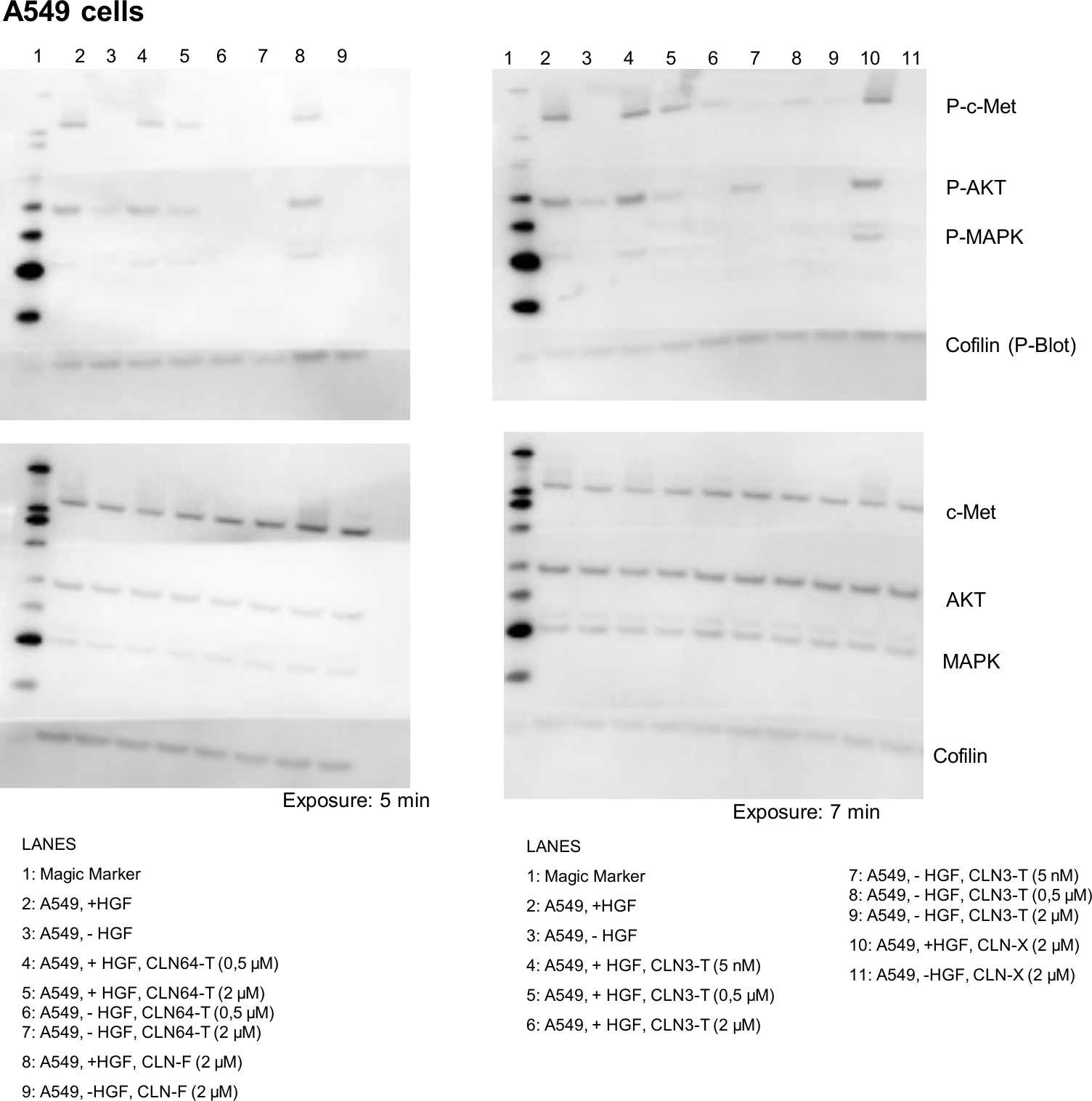

Supplement: S5 Fig — For each analysis the membrane for the detection of the phosphorylated protein (upper image) and the total protein (lower image) is shown. A549 cells treated with three concentrations of CLN64-T and CLN3-T and the control aptamers CLN-X and CLN-F. (TIF) [file pone.0142412.s005.tif]

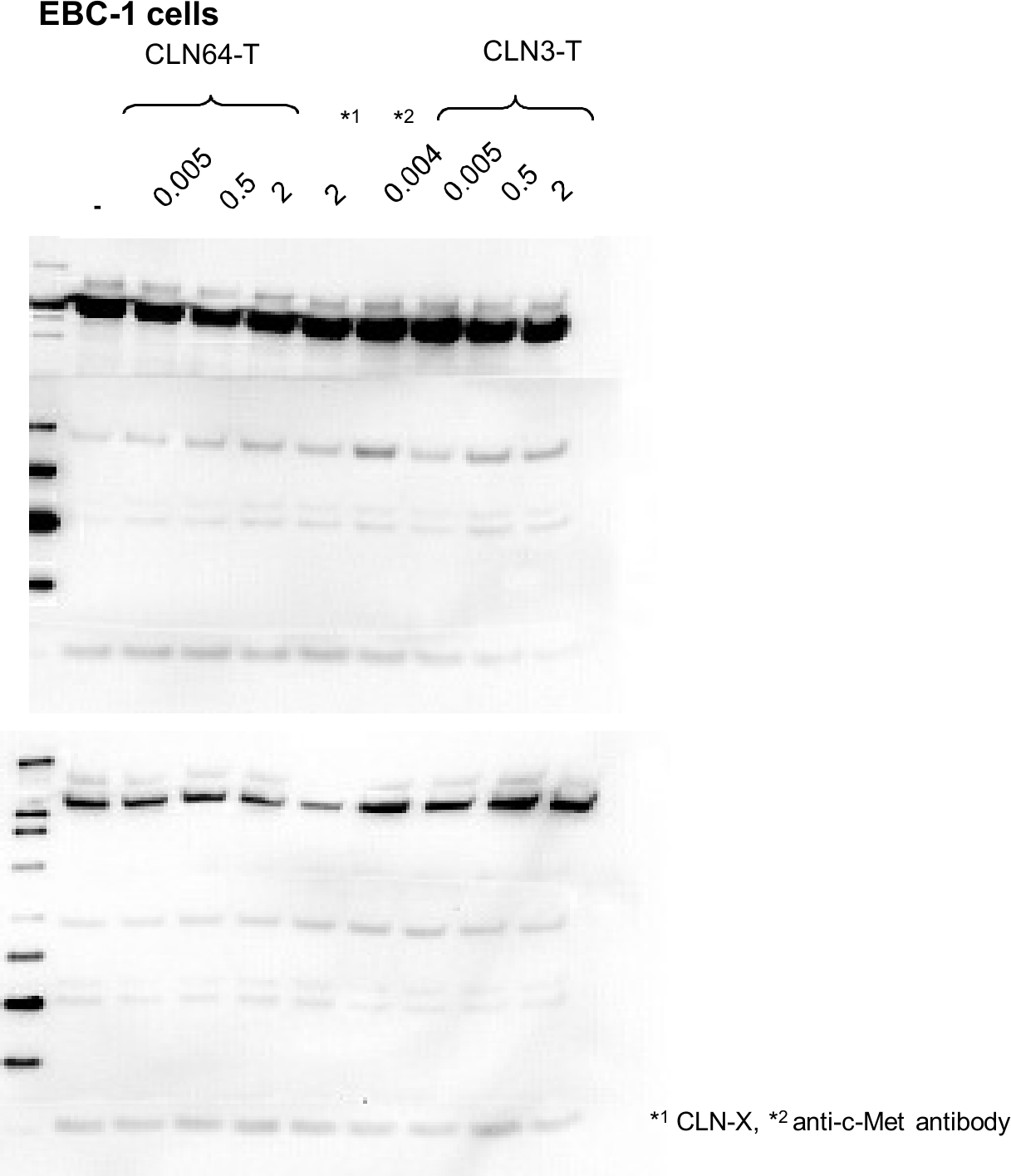

Supplement: S6 Fig — For each analysis the membrane for the detection of the phosphorylated protein (upper image) and the total protein (lower image) is shown. EBC-1 cells treated with three concentrations of CLN64-T and CLN3-T and the control aptamer CLN-X. (TIF) [file pone.0142412.s006.tif]

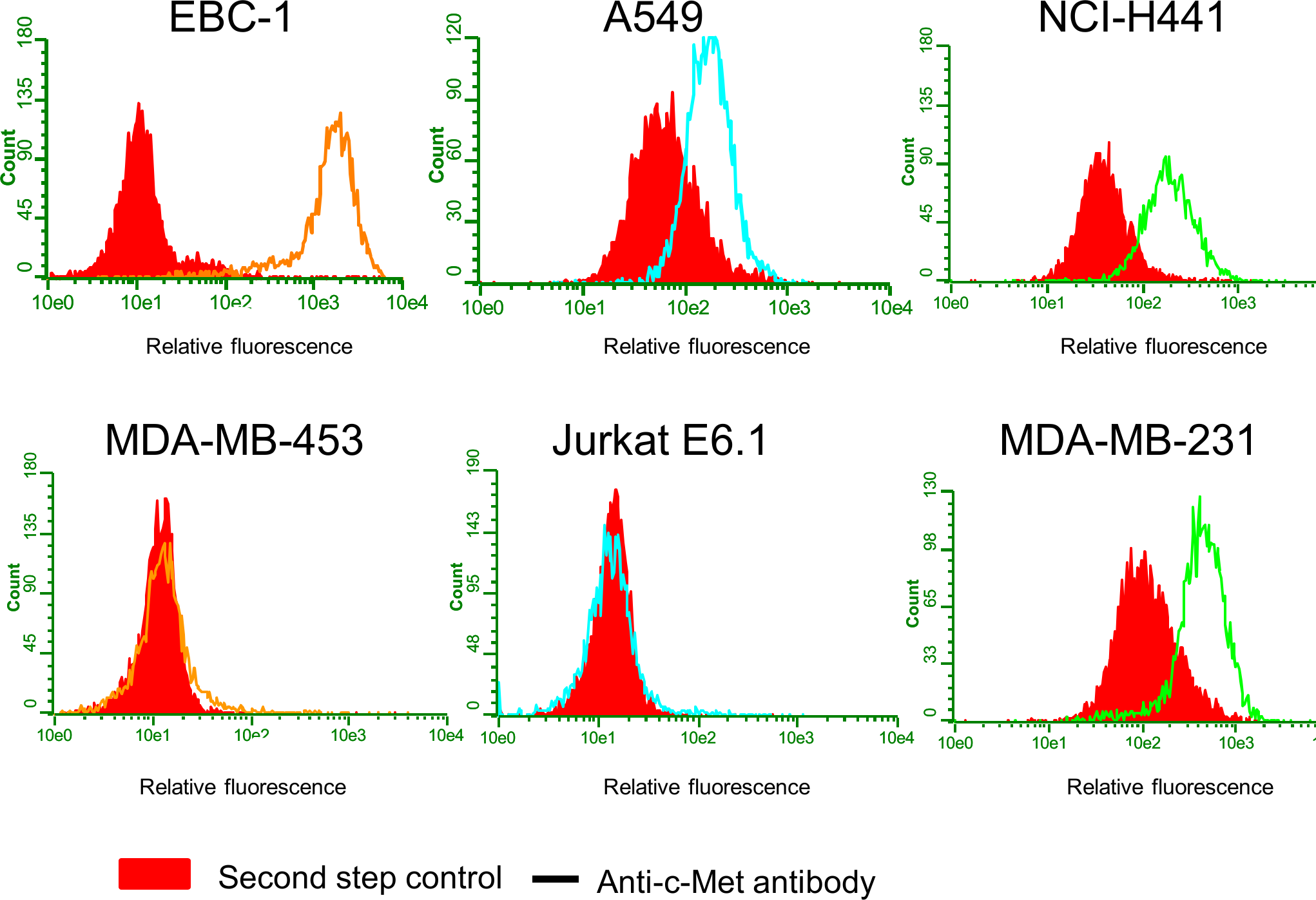

Supplement: S7 Fig — EBC-1, A549, NCI-H441, MDA-MB-453, Jurkat E6.1 and MDA-MB-231 cells were analyzed with anti-c-Met antibody at saturating concentrations. (TIF) [file pone.0142412.s007.tif]

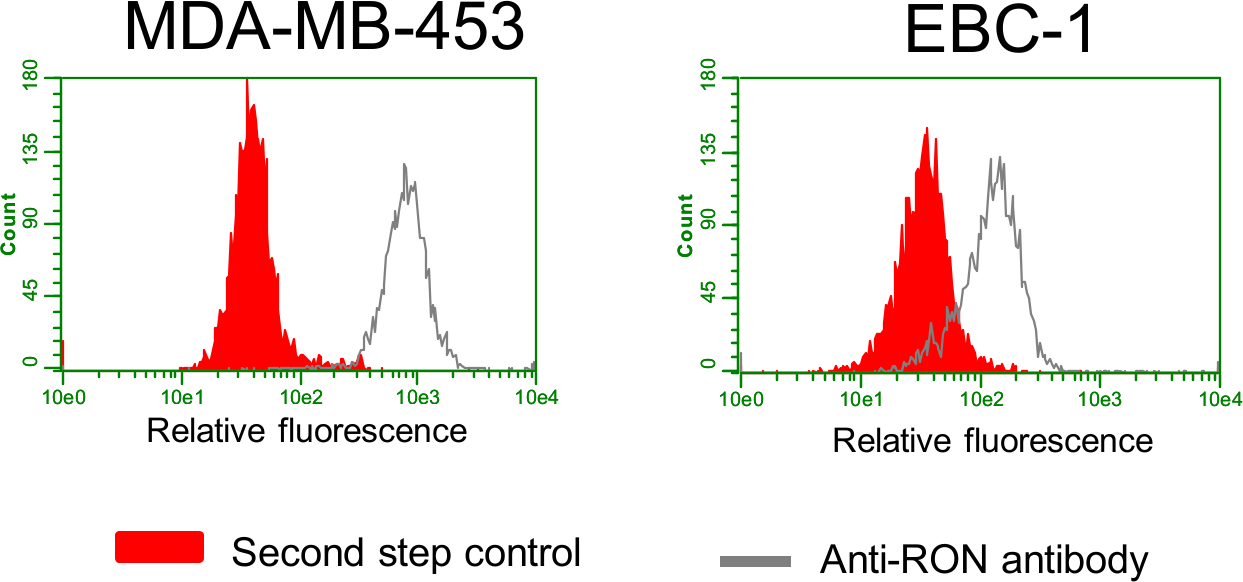

Supplement: S8 Fig — (TIF) [file pone.0142412.s008.tif]

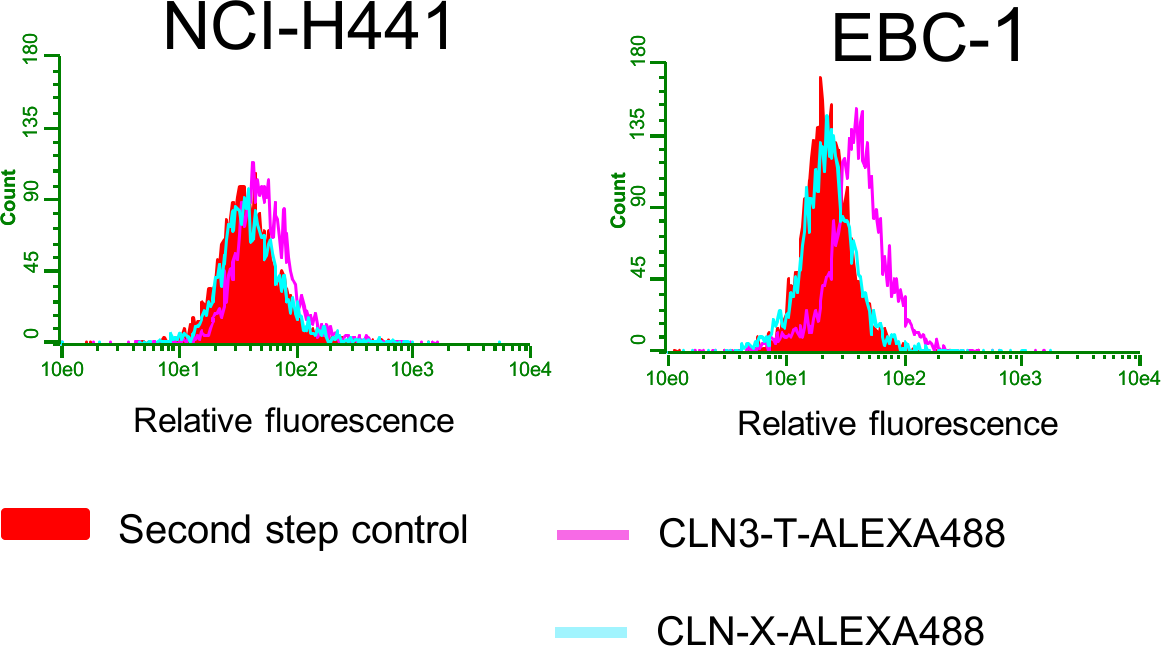

Supplement: S9 Fig — In comparison to the manuscript, c-Met specific aptamer CLN3-T and control aptamer CLN-X were directly labeled with ALEXA488. When comparing the signal on EBC-1 cells, the signal amplification with directly labeled ALEXA488 is lower than for detection with biotinylated aptamers and streptavidin phycoerythrin. Nevertheless CLN3-T shows a low signal for binding to NCI-H441 cells, which can likely be amplified by the use of biotinylated aptamers. (TIF) [file pone.0142412.s009.tif]

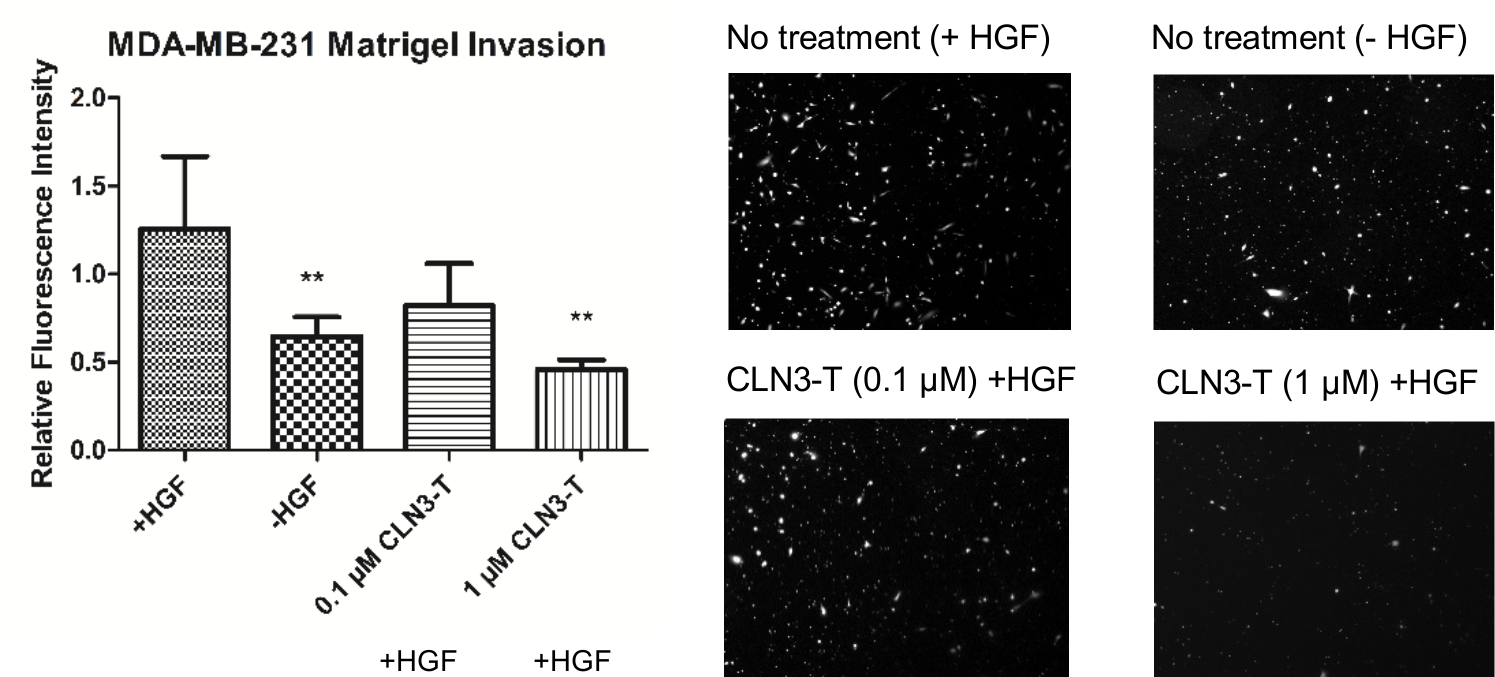

Supplement: S10 Fig — Matrigel cell invasion was determined by fluorescence measurements 485/530 nm (excitation/emission) (n = 3) at various concentrations of CLN3-T treatment. ** p < 0.05 versus the control group treated with HGF only. The invaded cells were also visualized by fluorescence microscopy (excitation: 488 nm) with a 10-fold objective. (TIF) [file pone.0142412.s010.tif]
